# A Disposable paper breathalyzer with an alcohol sensing organic electrochemical transistor

**DOI:** 10.1038/srep27582

**Published:** 2016-06-13

**Authors:** Eloїse Bihar, Yingxin Deng, Takeo Miyake, Mohamed Saadaoui, George G. Malliaras, Marco Rolandi

**Affiliations:** 1 Department of Bioelectronics, Ecole Nationale Supérieure des Mines, CMP-EMSE, MOC, 13541 Gardanne, France; 2 Microvitae Technologies, Hôtel Technologique, Europarc Sainte Victoire, Route de Valbrillant, Meyreuil, 13590, France; 3 Department of Flexible Electronics, Ecole Nationale Supérieure des Mines, CMP-EMSE, MOC, 13541 Gardanne, France; 4 Department of Materials Science and Engineering, University of Washington, Seattle, WA, 98195, USA; 5 Department of Electrical Engineering, University of California, Santa Cruz, CA, 95064, USA

## Abstract

Breathalyzers estimate Blood Alcohol Content (BAC) from the concentration of ethanol in the breath. Breathalyzers are easy to use but are limited either by their high price and by environmental concerns, or by a short lifetime and the need for continuous recalibration. Here, we demonstrate a proof-of-concept disposable breathalyzer using an organic electrochemical transistor (OECT) modified with alcohol dehydrogenase (ADH) as the sensor. The OECT is made with the conducting polymer poly(3,4-ethylenedioxythiophene):poly(styrenesulfonate) (PEDOT:PSS), and is printed on paper. ADH and its cofactor nicotinamide adenine dinucleotide (NAD^+^) are immobilized onto the OECT with an electrolyte gel. When the OECT-breathalyzer is exposed to ethanol vapor, the enzymatic reaction of ADH and ethanol transforms NAD^+^ into NADH, which causes a decrease in the OECT source drain current. In this fashion, the OECT-breathalyzer easily detects ethanol in the breath equivalent to BAC from 0.01% to 0.2%. The use of a printed OECT may contribute to the development of breathalyzers that are disposable, ecofriendly, and integrated with wearable devices for real-time BAC monitoring.

The euphoria from drinking alcoholic beverages makes them popular worldwide. Abuse in alcohol (ethanol) consumption leads to dependence, behavioral problems, and fatal accidents^1^. In 2013, 10,076 people lost their lives in alcohol-related-driving accidents in the United States alone, accounting for nearly 31% of all traffic related deaths^1^. Driving under the influence is illegal and the maximum allowed blood alcohol concentration (BAC) is 0.05–0.08%, in most countries^2^. A breathalyzer measures the concentration of ethanol in the breath to estimate the BAC of an individual. The first generation of breathalyzers uses a liquid dye sensitive to ethanol exposure, potassium dichromate, and a photodetector^3^. Reliability of these detectors is a challenge and potassium dichromate is environmentally toxic^3^. A new generation of breathalyzers uses the ethanol in the breath to power a fuel cell whose output is proportional to the ethanol concentration^4^. These breathalyzers are connected directly to smart phones to test one’s alcohol level before attempting to drive. However, these breathalyzers are still impractical because they require constant recalibration^4^. To-date the most reliable BAC tests and the only one that is admissible in court is the blood test, which is difficult to administer on site or for preventative purposes. A breathalyzer as easy to use as an inexpensive and disposable glucose paper-strip sensor would greatly simplify BAC testing^5^.

Organic electrochemical transistors (OECTs) are excellent candidates for disposable biosensors because they are inexpensive, they can be made on flexible substrates, and they can be printed on paper^6^,^7^. OECTs are typically made with poly(3,4-ethylenedioxythiophene) doped with poly(styrenesulfonate) PEDOT:PSS. PEDOT:PSS is a *p-*type organic semiconductor with several applications in bioelectronics^8^,^9^. Coupled with enzymes in the electrolyte, PEDOT:PSS OECTs are able to detect micro molar glucose concentration in human blood and sweat^10^. OECTs sensors are amenable to screen-printing^11^ and inkjet printing^12^ for rapid and inexpensive manufacturing. Here, we demonstrate an early stage proof-of-concept OECT-breathalyzer on paper by integrating a PEDOT:PSS OECT with the enzyme alcohol dehydrogenase. This proof-of-concept OECT-breathalyzer may aid the development of a breathalyzer that is easy-to-use, inexpensive, easily calibrated, and can be coupled with a cell-phone or a smart watch for BAC self-testing to reduce alcohol related traffic accidents.

## Results

**Figure 1:**
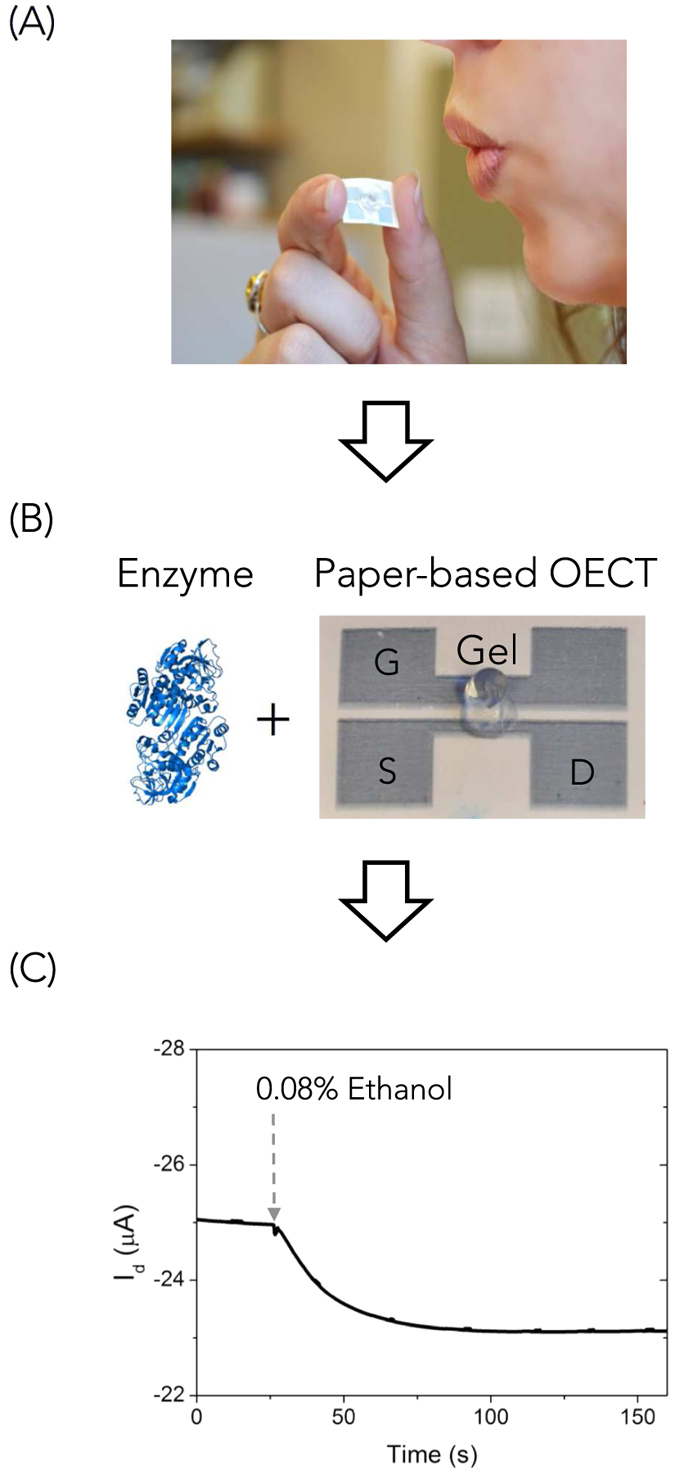
Concept of the OECT-breathalyzer. (**A**) Simply breathing on the printed PEDOT:PSS OECT allows for alcohol detection. (**B**) The alcohol dehydrogenase (ADH) enzyme and the OECT are the key components of the sensor. The OECT is printed on paper, and comprises a channel, source (S), drain (D), and gate (G) electrodes made of PEDOT:PSS. The enzyme electrolyte gel is deposited to the OECT bridging the channel and gate. (**C**) I_d_ response of the OECT upon exposure to ethanol.

The OECT is printed on a paper substrate that is approximately 1.5 cm × 1 cm and it is easy to handle. Breathing onto the device enables detection of ethanol concentration in the breath (Fig. 1A). The printed OECT on paper has planar geometry with channel, source (S), drain (D), and gate (G) electrodes made of PEDOT:PSS (Fig. 1B), a structure that is compatible with rapid, one-step fabrication of the device. We modify commercially available PEDOT:PSS to make it compatible with inkjet printing on paper (see Suppl. Info). A key challenge for the fabrication of an enzyme-based OECT sensor is the immobilization of the enzyme and its cofactor. In this work, the enzyme alcohol dehydrogenase (ADH) and its cofactor nicotinamide adenine dinucleotide (NAD^+^) are trapped in a collagen-based gel deposited onto the channel of the OECT (Fig. 1B). Exposing the OECT-breathalyzer to ethanol from the breath causes a marked decrease of the source-drain current, I_d,_ which is used as the output signal for detection (Fig. 1C).

**Figure 2:**
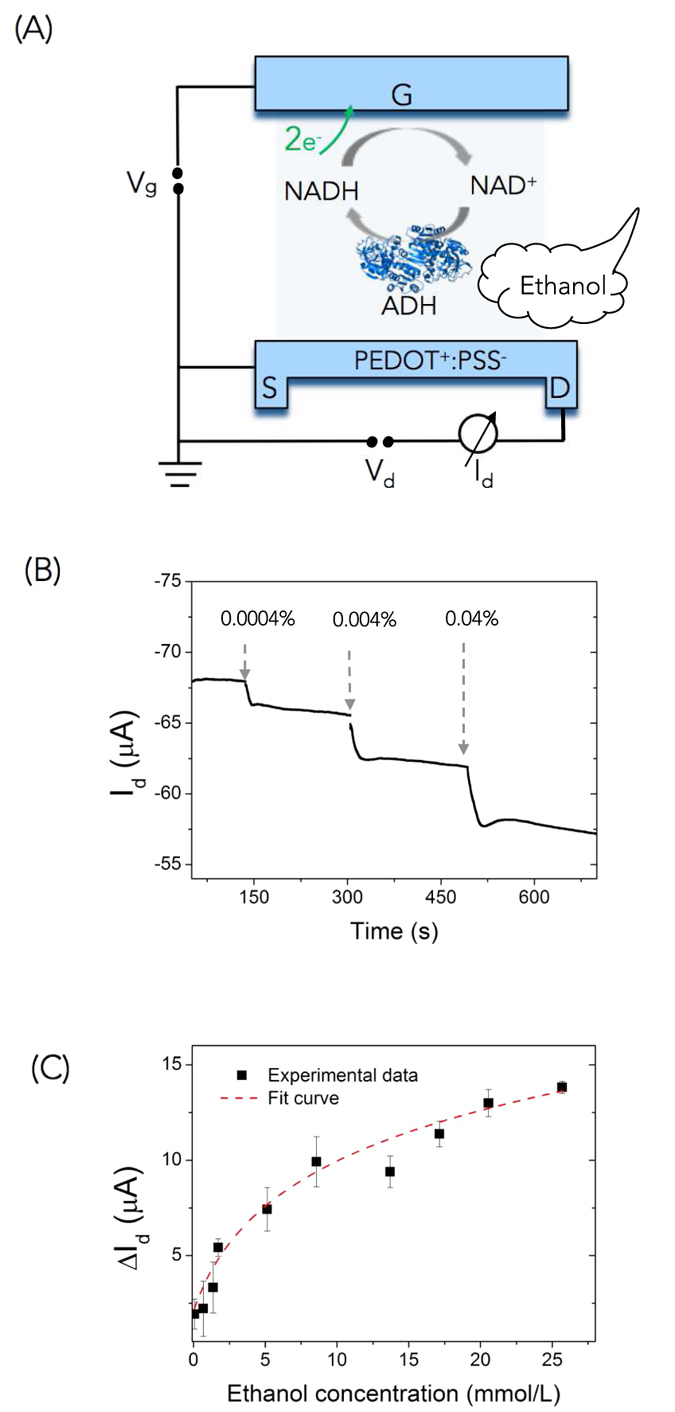
Alcohol detection in solution. (**A**) Enzymatic reaction of ethanol and ADH in electrolyte solution. Ethanol is oxidized to acetaldehyde, and NAD^+^ is reduced to NADH. (**B**) Step change in I_d_ when adding 0.0004%, 0.004%, and 0.04% ethanol solutions, with V_g_ = 0.5 V, V_d_ = −0.7 V. (**C**) Variation of I_d_ is plotted at different alcohol concentrations in solution. ΔI_d_ = (I_0_–I_d_), where I_0_ is I_d_ before exposure to ethanol. The dash line is a fit to equation (3). n = 3.

The decrease in I_d_ upon ethanol exposure arises from a series of chemical reactions that oxidize ethanol into acetaldehyde and produce electrons as byproducts, which in turn decrease the conductivity of the OECT channel (Fig. 2A).

First the reaction:


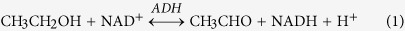


yields the reduced form of nicotinamide adenine dinucleotide (NADH), which itself oxidizes according to^13^:


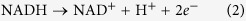


The electrons produced by the NADH oxidation are collected from the gate electrode of the OECT-breathalyzer (Fig. 2A), and cause a shift of the applied gate potential to the channel/electrolyte interface, leading to a decrease in I_d_^14^. I_d_ is written as^14^:


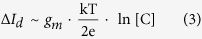


where *g*_*m*_ is the transconductance of the OECT and *C* is the concentration of the molecule contributing electrons to the gate (NADH in this case) (Figs S1 and S2). Since the concentration of NADH is directly related to the concentration of ethanol in the breath^15^,^16^,^17^, this mechanism leads to quantitative ethanol detection. This was confirmed by exposing the OECT-breathalyzer to a series of phosphate-buffered saline (PBS) solutions containing ADH, NAD^+^, and different amounts of ethanol. The results, shown in Fig. 2B, demonstrate that ethanol detection is achieved for concentrations as low as 0.0004%, and that higher ethanol concentration causes a bigger drop in I_d_. The I_d_ drop occurs in seconds with an immediate enzyme response and steady state is reached in seconds. As a control, in absence of ADH, there is no response from the device upon addition of different ethanol concentrations (Fig. S3). We calibrate the sensor to different concentrations (Fig. 2C). Higher ethanol concentration corresponds to higher ΔI_d_ as observed with NADH (Fig. S1). The response of the sensor is logarithmic with ethanol concentration, as expected from Eq. (3).

**Figure 3:**
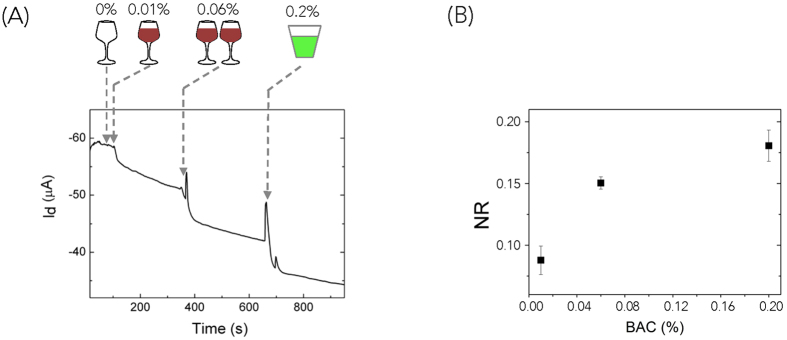
Alcohol detection in breath. (**A**) Step change in I_d_ when breathing 0%, 0.01%, 0.06%, 0.2% ethanol vapor on to the OECT, with V_g_ = 0.5 V, V_d_ = −0.7 V. Breath alcohol concentration is tested 30 minutes after drinking one glass of wine (0.01%) and two glasses of wine (0.06%). 0.2% breath ethanol vapor is from mouthwash, which contains 21.6% alcohol. The BAC is calibrated with a commercially available breathalyzer before testing on the device. (**B**) Normalized response (NR) of I_d_ is plotted at different breath alcohol concentrations (BAC). NR = (I_0_–I_d_)/I_0_. n = 3.

Finally, we demonstrate that the OECT-breathalyzer detects ethanol content in the breath of human subjects. For this demonstration, we use bovine gelatin to integrate the ADH and NAD^+^ onto the OECT, as breathing onto a liquid electrolyte causes excessive noise and makes packaging of the OECT-breathalyzer challenging. Ethanol in the breath is related to BAC by a factor of approximately 1/2100, which tends to vary with each individual^18^. Seven volunteers participate in the breath alcohol test for BAC detection. One volunteer serves as the control, while other volunteers consume different amounts (120 ml and 240 ml) of red wine (Les 3 filles, 2014, Merlot, 13% alcohol content). The experiment has been reproduced for each amount of wine with 3 volunteers. Thirty minutes after wine consumption, the volunteers are subject to a breath test with a commercial breathalyzer (Breathometer^TM^) for calibration and then are subject to the same test with the OECT-breathalyzer. Subsequently, the volunteer who serves as the control rinses their mouth with mouthwash (Listerine^®^, 21.6% ethanol content) and immediately takes another set of breath tests. The results from the two tests are compared for accuracy. The test from the volunteer serving as control (0% BAC) results in no response from the OECT-breathalyzer. In contrast, when the volunteers who have consumed wine take the test after one glass (0.01% BAC) and two glasses (0.06% BAC), an immediate response in I_d_ of the OECT-breathalyzer is observed (Fig. 3A). This response scales with the amount of alcohol consumed. The test corresponding to mouthwash registers an apparent BAC of 0.2%, consistent with the high alcohol concentration in this solution. (Fig. 3B). Similarly to commercial breathalyzers, the OECT-breathalyzer does not seem to be affected by variation in breathing time from different volunteers as indicated by the small spread in the data for the NR (Fig. 3B). We suggest that as long as a volunteer breathes on the OECT-breathalyzer long enough, the ethanol concentration on the device will equilibrate with the ethanol concentration in the breath. This proof-of-concept demonstrates that this simple OECT-breathalyzer is able to detect BAC in human subjects with performance comparable with a commercial breathalyzer.

## Discussion and Conclusions

We demonstrate the first alcohol sensor made with an organic electrochemical transistor integrated with the enzyme alcohol dehydrogenase and its cofactor. This OECT-breathalyzer is easy to fabricate using printing techniques, requires no metal deposition, and is made on an inexpensive, disposable, and biodegradable paper support. The use of a gel to immobilize the enzyme and its cofactor makes the device robust and easy to use. We show that the OECT-breathalyzer detects ethanol in both solution and vapor (such as breath). We conduct preliminary tests with a limited pool of human volunteers and compare the performance of the OECT-breathalyzer with the performance of a commercial breathalyzer. The OECT-breathalyzer is able to detect the consumption of just one glass of red wine. For further optimization, studies with a larger pool of human volunteers are required. This work may help develop alcohol sensors that are easy to integrate with portable/wearable electronics such as smartphones and smart watches. These devices could, in turn, prevent a vehicle to start if they detect the driver to be under the influence and therefore reduce alcohol-associated traffic accidents.

## Materials and Methods

### PEDOT:PSS ink

The PEDOT:PSS ink consists of the commercially available PEDOT:PSS (Heraeus, Clevios PH1000) dispersion with 20 wt% ethylene glycol (Sigma Aldrich) and a combination of organic solvents. We add 0.8 wt% glycidoxypropyltrimethoxysilane (GOPS, Sigma Aldrich) to the ink to prevent delamination, and 0.3% surfactants to match the surface tension of the ink with the substrate.

### Ink-jet printing

We use a Dimatix DMP-2800 inkjet printer to print the OECT onto a coated paper (Arjo Wiggins, Inc.). In this study, two layers of PEDOT:PSS are deposited for a total thickness of 190 nm. The printed device is cured in a conventional oven at 160 °C for 30 min. The dimensions of the channel are 1 × 5 mm^2^ and 2 × 5 mm^2^ for the gate (Fig. 1). For the alcohol solution measurements, 9 mM NAD^+^ (Sigma Aldrich) are mixed in 0.1 M standard phosphate buffer solution (PBS, Sigma Aldrich). For each measurement, 1.5 mg/mL ADH (Sigma Aldrich) in PBS is added to the in NAD^+^ and PBS mix at a 1:10 ratio of the total volume. The pH of the electrolyte is adjusted to 8.2 and measured with a pH meter to meet the requirements of the enzyme. A PDMS well is attached to the OECT to confine the electrolyte, defining an active device area of 4 × 4 mm^2^. The well is filled with 20 μL electrolyte. For the alcohol vapor measurements, we formulate a 2 wt% bovine gelatin (Sigma Aldrich) in PBS, containing the same proportion of ADH and NAD^+^ as in the liquid electrolyte above. 15 μL gel solution is drop-casted onto the device and cured at 4 °C for 30 min to form gel.

### OECT Electrical Characterization

Electrical characterization is conducted with an Agilent 4155C semiconductor parameter analyzer. During the experiment, V_g_ varies from 0 to 0.7 V and the V_d_ from −0.8 to 0 V. For the measurements, we apply constant V_g_ = 0.5 V and V_d_ = −0.7 V respectively. Soft carbon electrodes connect the OECT contacts and the Agilent. We prepare different concentrations of ethanol solution in DI water: 0.0004 wt%, 0.004 wt%, 0.008 wt%, 0.01 wt%, 0.03 wt%, 0.04 wt%, 0.08 wt%, 0.1 wt%, 0.12 wt%, 0.15 wt% and measure the response of the device for each concentration three times. During the alcohol solution tests (Fig. 2), we first wait for 120 s until the source drain current (I_d_) stabilizes after the application of bias on the OECT, and then add 2 μL of ethanol solution to the electrolyte at a 1:10 ratio. For the BAC tests (Fig. 3), we replace the electrolyte solution with gel on top of the OECT. Volunteers consuming different quantities of alcoholic beverages first have their BAC tested with a commercial breathalyzer: The Original Breathometer (Breathometer, Inc.), and then breathe onto the OECT-breathalyzer for a certain duration. The experiment has been tested three times for each concentration with volunteers. The methods were carried out in accordance with the approved guidelines, and all experimental protocols were approved by the direction of research of the Ecole des Mines de St. Etienne. All volunteers provided informed signed consent to participate in the study.

## Additional Information

**How to cite this article**: Bihar, E. *et al*. A Disposable paper breathalyzer with an alcohol sensing organic electrochemical transistor. *Sci. Rep.* **6**, 27582; doi: 10.1038/srep27582 (2016).

## Supplementary Material

Supplementary Information
