# Supplementary material for: A Disposable paper breathalyzer with an alcohol sensing organic electrochemical transistor
Source: Sci Rep. 2016 Jun 13;6:27582. doi: 10.1038/srep27582 (PMC4904368; doi:10.1038/srep27582)
Supplement: Supplementary Information [file srep27582-s1.doc]

Supplementary Materials

**A Disposable paper breathalyzer with an alcohol sensing organic electrochemical transistor**

Eloїse Bihar, Yingxin Deng, Takeo Miyake, Mohamed Saadaoui, George G. Malliaras, and Marco Rolandi


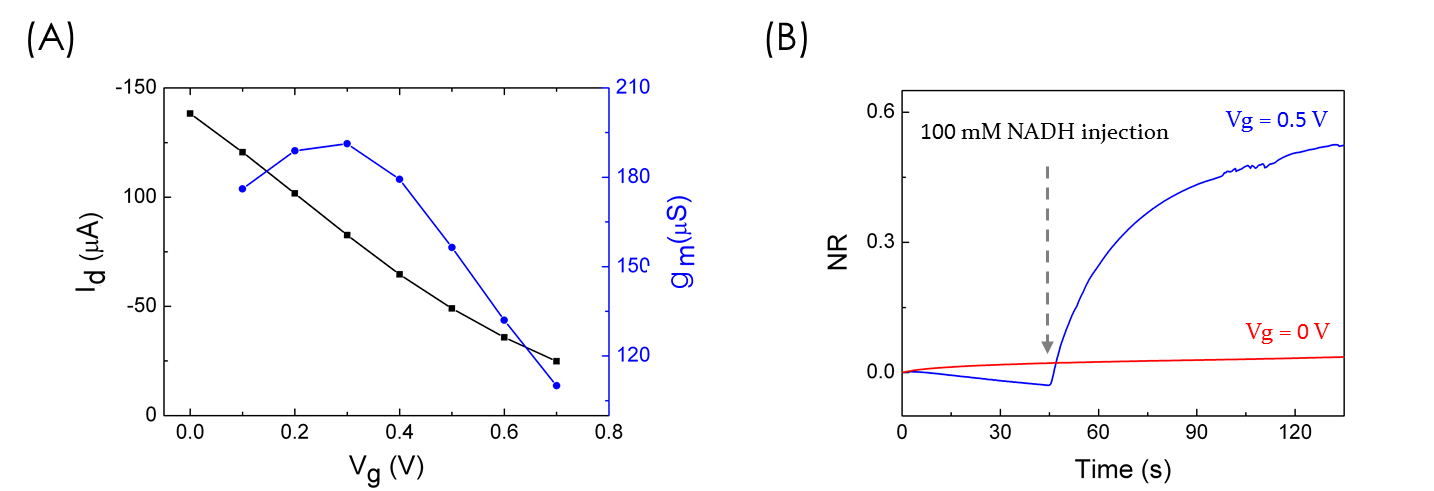


**Fig. S1.** **PEDOT:PSS OECT alcohol sensor characteristics.** **(** **A)** Transfer curve and resulting transconductance of OECT with 0.2wt% bovine gel at V_d_ = -0.7 V. **(B)** Normalized response (NR) of I_d_ is plotted at V_g_ = 0 V and V_g_ = 0.5 V. Arrows indicate the addition of 100 mM NADH. NR= (I_0_- I_d_)/I_0_, where I_0_ is I_d_ before exposure to NADH.


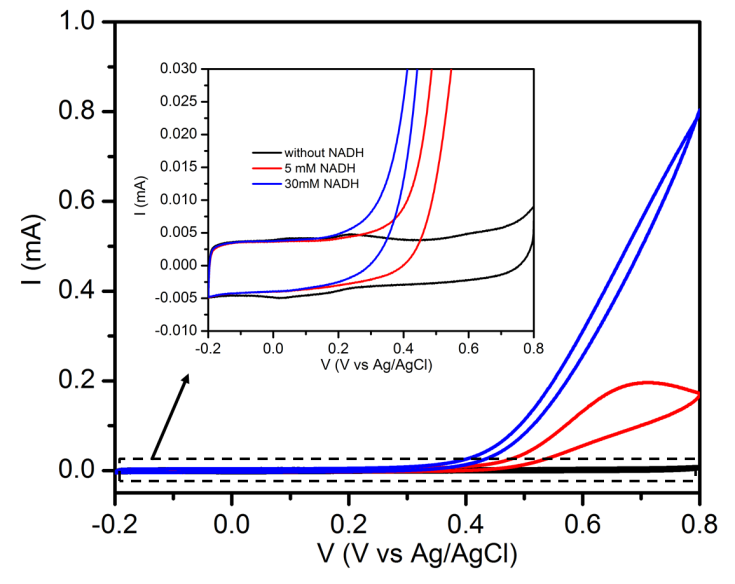


**Fig. S2.** **NADH oxidation at PEDOT:PSS electrode cycled at 10 mV/s.** Oxidation starts for V= 0.3 V


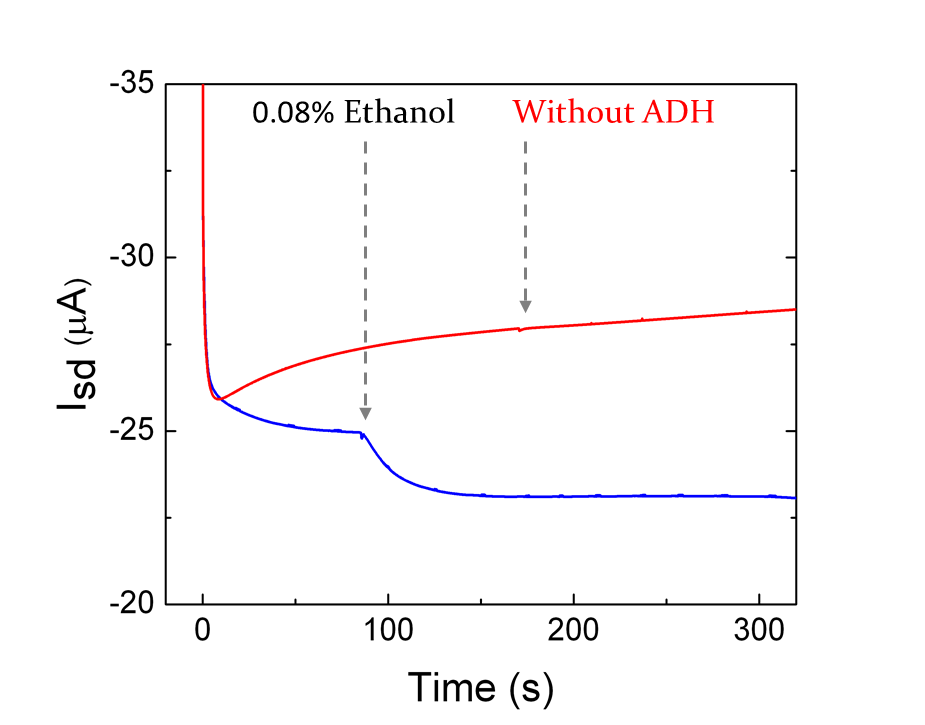


**Fig. S3.** **Response of OECT to ethanol.**  When ethanol is added to an OECT without the enzyme ADH no change in I_d_ is detected, while when ADH is present I_d_ drops as expected. The initial drop around 0 s corresponds to equilibration of the sensor.
